# Langmuir–Blodgett Films with Immobilized Glucose Oxidase Enzyme Molecules for Acoustic Glucose Sensor Application

**DOI:** 10.3390/s23115290

**Published:** 2023-06-02

**Authors:** Ilya Gorbachev, Andrey Smirnov, George R. Ivanov, Tony Venelinov, Anna Amova, Elizaveta Datsuk, Vladimir Anisimkin, Iren Kuznetsova, Vladimir Kolesov

**Affiliations:** 1 Kotelnikov Institute of Radio Engineering and Electronics of RAS, 125009 Moscow, Russia; iliyagor36@gmail.com (I.G.); andre-smirnov-v@yandex.ru (A.S.); datsuk.er@phystech.edu (E.D.); anis@cplire.ru (V.A.); kvv@cplire.ru (V.K.); 2 University Laboratory “Nanoscience and Nanotechnology”, University of Architecture, Civil Engineering and Geodesy, 1164 Sofia, Bulgaria; george@at-equipment.com (G.R.I.); tvenelinov_fhe@uacg.bg (T.V.); anna.amova@gmail.com (A.A.)

**Keywords:** Langmuir–Blodgett films, Langmuir monolayers, enzymatic biosensors, acoustoelectronic glucose sensor

## Abstract

In this work, a sensitive coating based on Langmuir–Blodgett (LB) films containing monolayers of 1,2-dipalmitoyl-sn-glycero-3-phosphoethanolamine (DPPE) with an immobilized glucose oxidase (GOx) enzyme was created. The immobilization of the enzyme in the LB film occurred during the formation of the monolayer. The effect of the immobilization of GOx enzyme molecules on the surface properties of a Langmuir DPPE monolayer was investigated. The sensory properties of the resulting LB DPPE film with an immobilized GOx enzyme in a glucose solution of various concentrations were studied. It has shown that the immobilization of GOx enzyme molecules into the LB DPPE film leads to a rising LB film conductivity with an increasing glucose concentration. Such an effect made it possible to conclude that acoustic methods can be used to determine the concentration of glucose molecules in an aqueous solution. It was found that for an aqueous glucose solution in the concentration range from 0 to 0.8 mg/mL the phase response of the acoustic mode at a frequency of 42.7 MHz has a linear form, and its maximum change is 55°. The maximum change in the insertion loss for this mode was 18 dB for a glucose concentration in the working solution of 0.4 mg/mL. The range of glucose concentrations measured using this method, from 0 to 0.9 mg/mL, corresponds to the corresponding range in the blood. The possibility of changing the conductivity range of a glucose solution depending on the concentration of the GOx enzyme in the LB film will make it possible to develop glucose sensors for higher concentrations. Such technological sensors would be in demand in the food and pharmaceutical industries. The developed technology can become the basis for creating a new generation of acoustoelectronic biosensors in the case of using other enzymatic reactions.

## 1. Introduction

The measurement of sugar concentration in liquid solutions is an important task for many fields of science and technology. The control of this parameter is important in the production of food beverages, pharmaceuticals, cosmetics, plastics, and tobacco products [1,2,3,4,5]. In addition, blood glucose levels are an important indicator of human health. By controlling their measurement, it is possible to determine the presence of diabetes, as well as many diseases associated with metabolic disorders [6,7]. Almost all modern glucometers operate using the electrochemical principle. It is based on measuring the change in electrophysical parameters resulting from the interaction of a sugar-containing liquid with a specific reagent deposited on the electrode structure of the glucometer [8]. Glucometers based on the use of calorimetric and optical methods are also known [9,10,11]. In recent years, noninvasive glucometers based on the indirect measurement of blood glucose concentration have been actively developed. In this case, the parameters of biological fluids are measured: sweat, urine, saliva, or the blood filling of blood vessels [12,13,14,15,16,17].

It should be noted that, despite the great demand for such devices, there are still problems associated with increasing the reliability, sensitivity, and repeatability of their results. Machine learning technologies can be used to solve these problems [18]. This will make it possible to predict the dynamics of the course of the pathology. This is important in the development of systems for the automatic dosing of drugs. Another solution to these problems is the creation of enzymatic biosensors. These sensors have a high sensitivity and selectivity to target molecules [19,20]. This approach is applicable, among other things, to the creation of wearable multisensor systems [21,22], as well as systems for the continuous monitoring of glucose levels [23].

The immobilization of enzyme molecules on a substrate is one of the main tasks in the development of enzymatic biosensors. There are several ways to create sensor coatings with immobilized enzyme molecules. It is possible to use such methods as linker bonds with epoxy groups, fixation in various hydrogels, biopolymers, polyelectrolytes, etc. [24,25,26,27,28,29,30]. It should be noted that in this case the enzyme is located inside the matrix and this complicates access to it by the test liquid. This leads to a decrease in the sensitivity of the sensor and a decrease in its reliability. The problem of increasing the lifetime and stability of the enzyme is important due to the need to create systems for monitoring changes in glucose levels over a long time [31,32].

Langmuir–Blodgett (LB) technology can be used to solve these problems. This technology makes it possible to form a highly ordered monolayer of surfactant molecules at the water–air interface with a simultaneous immobilization of enzyme molecules in this monolayer. The successive transfer of such monolayers onto solid substrates makes it possible to form sensor coatings whose structures reproduce an element of the microbial cell membrane. In this case, the outer monolayers of the film will perform the function of protecting the enzyme from environmental influences. It should be noted that, as in the case of the immobilization of enzymes in films, there is a possibility of reducing the sensitivity of the created coating due to the presence of a layer of close-packed surfactant molecules covering the enzyme [33,34]. However, to overcome this drawback, it is possible to immobilize the enzyme in an LB film with a heterogeneous morphology, for example, by creating a mixed film based on a monolayer of surfactant molecules of different types or a film with incorporated nanocarbon structures [35]. In this regard, an urgent task is to study the process of the immobilization of enzyme molecules in mixed Langmuir monolayers of surfactant molecules of various types. The process of the immobilization of glucose oxidase enzyme molecules was studied in [33,36]. The influence of the charge of the head group of surfactant molecules, the type of surfactant molecules, and the length of the hydrocarbon radical on the process of adsorption of enzyme molecules were also studied [37,38,39].

It should be noted that LB films can be used as a sensor layer for sensors of various types. For example, an LB film of tyrosine hydroxylase was used to create an electrochemical biosensor for drug compounds [40]. A Langmuir film based on a monolayer of ammonium octadecyltrimethyl and Prussian blue with an immobilized glucose oxidase enzyme was used to create an amperometric glucometer [41]. Mixed Langmuir films of polyaniline and stearic acid with immobilized cholesterol oxidase were proposed to be used to create an electrochemical cholesterol biosensor [42]. An electrochemical sensor for penicillin based on a mixed Langmuir monolayer of penicillinase–DMPA incorporated with carbon nanotubes was proposed in [43]. An LB film based on octadecylamine with an immobilized phenol oxidase enzyme was used to create an electrochemical biosensor for the fruit browning enzyme (phenol) [44]. Along with typical electrochemical, impedance, and optical glucose sensors, LB films containing enzyme molecules can be used to fabricate efficient acoustic sensors.

The principle of their operation is based on recording the characteristics of acoustic waves in piezoelectric structures that are in contact with the sensor film. Due to changes in the electrical and/or acoustic properties of the film as a result of biospecific interaction with the analyzed liquid, the phase and amplitude of the used acoustic wave change [45,46]. These changes can be easily detected by existing devices and using acoustic technology for studying polydisperse liquids of various compositions [47,48,49,50]. One of the advantages of acoustoelectronic technologies is the absence of contact between the substance under study and the electrode structure, in contrast to the electrochemical principle of biosensor implementation. Moreover, the advantages of the acoustoelectronic method include the possibility of cleaning the surface of the sensor coating from nonspecifically bound protein molecules using an acoustic stream [51].

It is necessary to clarify the question of the influence of LB films on the properties of acoustic waves to use them as sensor coatings. The influence of LB films on the characteristics of devices based on surface and bulk acoustic waves [52,53,54], Love waves [55], FBAR [56], and HF SAW resonators [57] was studied. As a result, it was shown that these films have almost no effect on the characteristics of acoustic waves of various types and can be effectively used as sensor coatings. It was shown that these films and acoustic devices can be used to create various gas sensors [57,58,59,60].

As for biological sensors, it was proposed in [61] to use the immobilization of a specific phage in an LB film deposited on the surface of a quartz microbalance. As a result, the possibility of registering β-galactosidase from *Escherichia coli* was demonstrated. Works on the study of the possibility of the immobilization of various enzymes in LB films and the creation of biosensors on this basis are currently at the initial stage of development and may lead to the creation of a new generation of biosensors [53,62].

In this article, a technology for immobilizing the enzyme glucose oxidase (GOx) into bilayer membrane-like LB films based on phospholipid molecules 1,2-dipalmitoyl-sn-glycero-3-phosphoethanolamine (DPPE) was developed. The morphology of the resulting LB films and their sensory properties to glucose solutions of various concentrations were studied.

## 2. Materials and Methods

### 2.1. Formation of a Sensitive Coating Based on the LB Film of Phospholipid Molecules with Immobilized Molecules of the GOx Enzyme

Powder of 1,2-dipalmitoyl-sn-glycero-3-phosphoethanolamine (DPPE) (Sigma-Aldrich, St. Louis, MO, USA, 99%) was dissolved in chloroform (Sigma-Aldrich, St. Louis, MO, USA, 99%) to obtain a solution with a concentration of 10^−3^ M/L.

It is known that GOx molecules are selectively sensitive to glucose [63]. In this work, a solution of the GOx enzyme in distilled water was used as an aqueous subphase. The enzyme concentration was 0.015 mg/mL. As is known, the DPPE phospholipid retains a neutral charge at pH values in the range of 5 to 7 [64]. The shift of the pH value to the acidic region allows one to polarize the DPPE molecule, creating an excess positive charge in the hydrophilic part of the molecule. In this regard, to increase the efficiency of GOx enzyme adsorption by a Langmuir DPPE monolayer, it is necessary to change the surface charge of its molecules from positive to negative. It is known that the GOx molecule has an isoelectric point at a pH of 4.2 [65], so the pH of the aqueous subphase was 4. A sodium acetate buffer solution was used to achieve such a pH. This solution was prepared by mixing aqueous solutions of sodium acetate (*CH_3_COONa*) and acetic acid (*CH_3_COOH*) with molar concentrations of 0.2 M/L and a volume ratio of *CH_3_COONa*:*CH_3_COOH* equal to 18:82. The resulting buffer solution was mixed with an aqueous subphase to obtain pH = 4. The pH value was controlled using a pH meter, pH-150MI (IzmTeh, Moscow, Russia).

**Figure 1:**
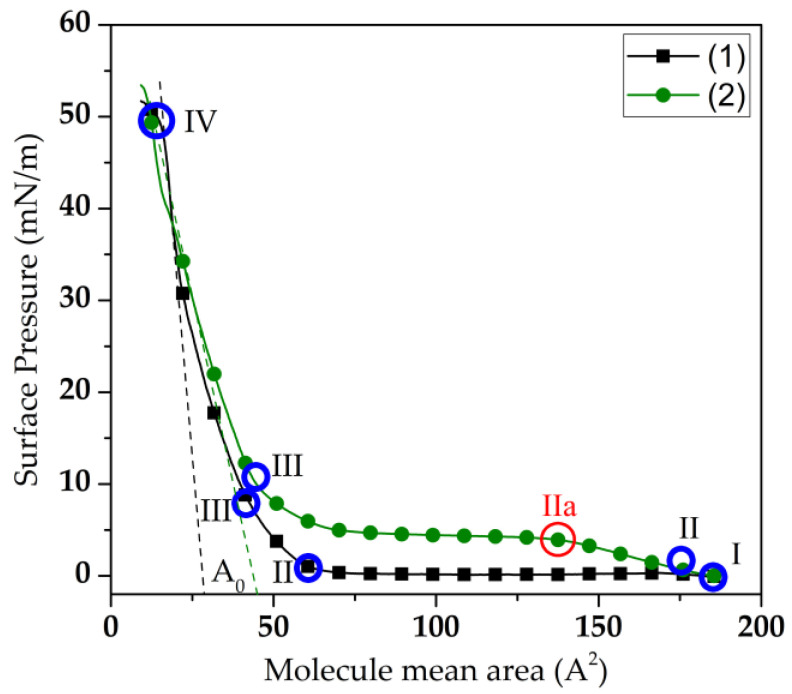
π-A isotherms of the Langmuir monolayer of DPPE molecules formed in the absence of dissolved GOx enzyme molecules in the aqueous subphase (1) and in their presence (2). In regions of compression isotherms I-II, II-III, and III-IV, the monolayer was in the gas, liquid, and condensed phases, respectively. IIa is additional phase transition point.

The formation of Langmuir monolayers and LB films was carried out on a KSV Nima LB Trough KN2001 setup (Nima KSV, Espoo, Finland) with a working surface area of 243 cm^2^. A solution of DPPE in chloroform was applied to the surface of the aqueous subphase with an aliquot volume of 50 µL. After 120 min from the moment the solution was applied to the water surface, the monolayer was compressed by movable barriers at a constant area loss rate of 0.7 cm^2^/min. The dependence of the surface pressure in the monolayer on the area occupied in it by one DPPE molecule (π-A isotherm) was recorded automatically using a Wilhelmy balance sensor. Figure 1 shows π-A isotherms of Langmuir DPPE monolayers formed on an aqueous subphase in the absence of dissolved GOx enzyme (1) and in its presence (2).

The specific area per DPPE molecule in the condensed phase (*A*_0_) and the compression modulus (*k*) of the Langmuir monolayer were determined using the obtained π-A isotherms [66]. The *A*_0_ value was determined using a tangent drawn to the π-A section of the III-IV isotherm corresponding to the condensed phase of the monolayer. The compression modulus was calculated by (1):

(1) $$k=-A_{0}\frac{d\pi }{dA}\;,$$

where the ratio *d*π/*dA* is numerically equal to the tangent of the slope of the tangent drawn to section III–IV of the π-A monolayer isotherm (Figure 1).

**Figure 2:**
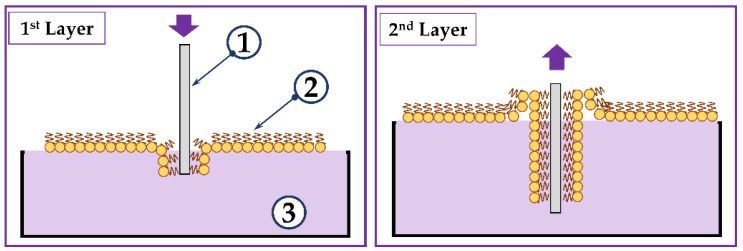
Schematic representation of the process of formation of the LB film of DPPE with immobilized GOx enzyme. 1, 2, and 3 are piezoelectric plate, monolayer of AA with immobilized GOx, and water subphase, respectively.

The transfer of monolayers to solid substrates was carried out using the Langmuir-Blodgett method. This method was applied for the production of sensitive films on the surfaces of acoustic devices described in detail in [39]. Lithium niobate (*LiNbO_3_*) polished on both sides was used as a substrate. The monolayer was compressed by movable barriers until a surface pressure of 40 mN/m was reached. Next, the substrate oriented perpendicularly to the water–air interface passed through the monolayer with the immobilized GOx enzyme at a speed of 1 mm/min. After complete immersion, the substrate remained under the water for 30 s. Next, the substrate was pulled out from under the water at a rate of 1 mm/min. Thus, a bilayer membrane-like DPPE film with immobilized GOx enzyme molecules was formed on both surfaces of the *LiNbO_3_* plate. The resulting sensitive coating dried for 4 h under a hood. Schematically, the process of Langmuir–Blodgett film formation is shown in Figure 2.

After the completion of the coating drying process, the side of the substrate with deposited interdigital transducers (IDTs) was cleaned from the DPPE film using chloroform (Sigma-Aldrich, St. Louis, MO, USA, 99%) and ethanol (Sigma-Aldrich, St. Louis, MO, USA, 95%). Thus, the sensor film remained only on the side of the plate free from the IDT.

### 2.2. Production of an Acoustic Delay Line

**Figure 3:**
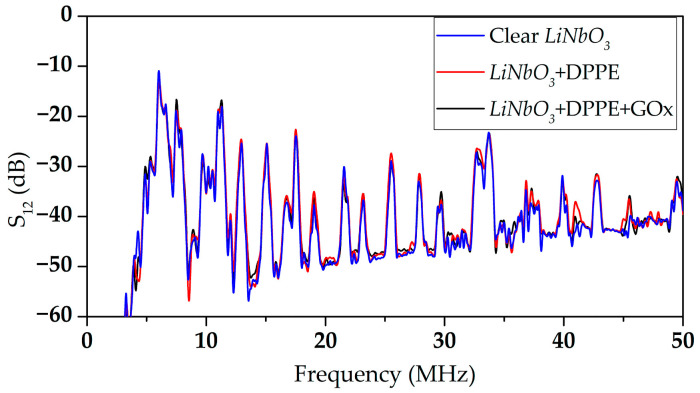
The frequency dependences of the parameter *S*_12_ of the fabricated acoustic delay line without LB film (blue line), with LB DPPE film (red line), and with LB DPPE film (black line) with immobilized GOx enzyme.

An acoustic delay line (DL) was fabricated on a YX *LiNbO_3_* plate polished on both sides with a thickness of 320 µm and 23.5 mm × 13.5 mm in size. Interdigital transducers (IDTs) were formed on the plate surface by magnetron sputtering and projection photolithography. The wavelength specified by the period of the IDT, the aperture, and the distance between the IDTs were 660 μm, 8 mm, and 10 mm, respectively. The operating frequencies of the formed DL were in the range of 1 to 50 MHz. The frequency dependences of the *S*_12_ parameter of the manufactured acoustic delay line without a LB film, with a LB film, and with a LB film with immobilized GOx enzyme are shown in Figure 3.

**Figure 4:**
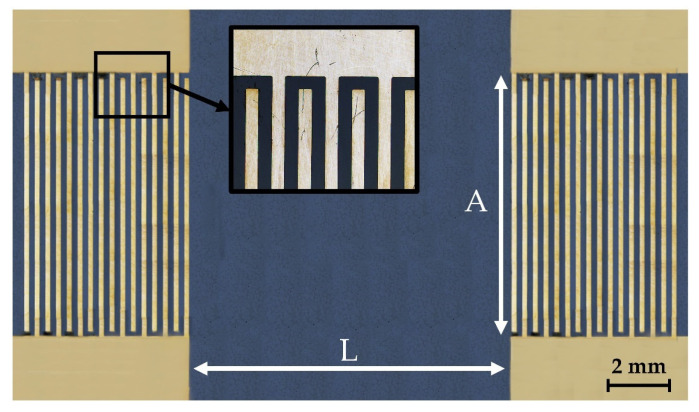
Photograph of the manufactured acoustic delay line. A and L are equal to 8 mm and 10 mm, respectively.

Figure 4 shows a photograph of the manufactured acoustic delay line.

### 2.3. Setup and Methods for the Study of Glucose-Sensitive Properties of the LB Film

**Figure 5:**
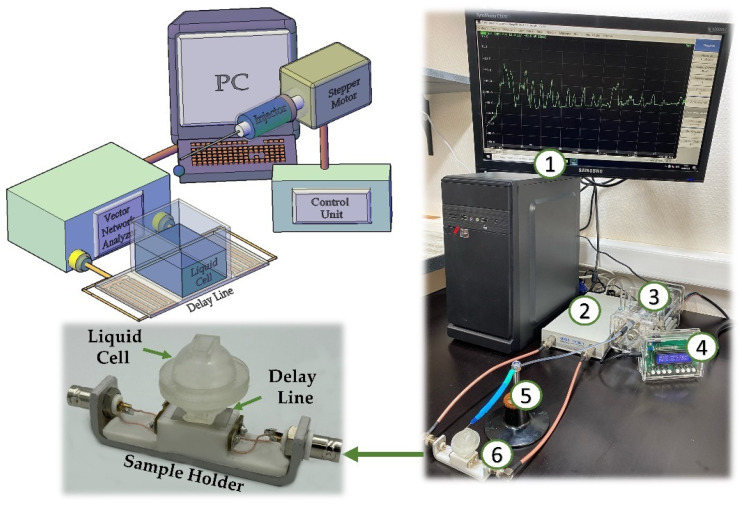
Scheme (**left**) and photo (**right**) of the setup for studying the sensory properties of the created LB DPPE film with the immobilized GOx enzyme deposited on the DL. The setup consists of a PC (1); a two-port vector network analyzer (2); an automated system for supplying a solution containing glucose (3, 4, 5); and an acoustic DL with an installed liquid cell (6).

A special experimental setup was designed and manufactured to study the sensory properties of the created LB DPPE film with the immobilized GOx enzyme (Figure 5).

The experimental setup for studying the sensory properties of the created LB DPPE film with an immobilized GOx enzyme deposited on the DL consisted of a PC (1) with an installed control program for a vector network analyzer (2) and an automatic glucose solution supply system (3,4,5). A two-port vector network analyzer Obzor TR1300/1 (Planar, Chelyabinsk, Russia) with an operating frequency range from 0.3 to 1300 MHz was used to measure the characteristics of acoustic waves. The created acoustic DL (6) was connected to the vector network analyzer via phase-stable cables. The sensitive film was formed on the side of the LZ free from IDTs. A cell with the liquid under study was placed between two IDTs on the side with the applied sensitive film. The cell was fabricated by DLP (Digital Light Processing) printing on an Anycubic PhotonS photopolymer printer (Anycubic, Shenzhen, China). The photopolymer resin Anycubic Basic (Anycubic, Shenzhen, China) was used for printing. After fabrication, the cell was washed in isopropyl alcohol to remove residual unpolymerized photopolymer resin and subjected to UV drying for 30 min. The cell edges were treated with salicylic acid phenol ester to ensure waterproofing. The automatic glucose solution supply system consisted of a frame with a dosing syringe (3), a stepper motor, and a microcontroller (4) to control the solution supply rate. The solution was supplied through a needle with a diameter of 0.1 mm (5). The speed of the syringe piston was 2.08 mm/h. At the indicated speed, a drop of 10 µL was formed at the end of the needle for 5 min and placed in the cell. Thus, there was a controlled change in the concentration of glucose solution in the cell.

**Figure 6:**
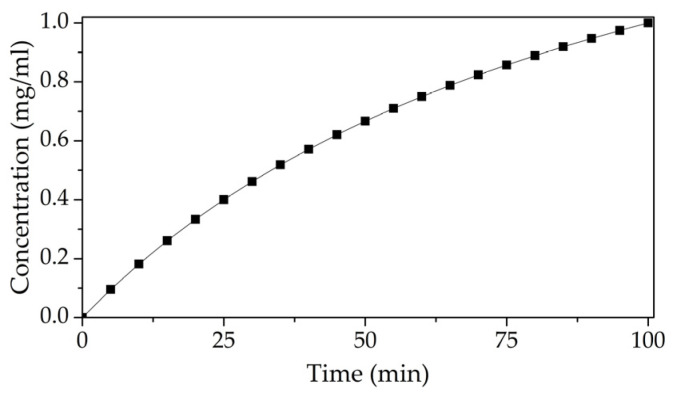
Time dependence of the concentration of an aqueous solution of glucose during the experiment.

During measurements, the cell was filled with distilled water (200 μL). With the indicated volume of liquid, a further increase in the height of the water column did not lead to a change in the amplitude-frequency characteristic (AFC) of the acoustic DL. A needle was placed above the hole located in the lid of the cell. This lid is used for delivering a glucose solution with a concentration of 2 mg/mL drop by drop into the cell. Thus, the glucose concentration in the measuring cell varied in the range from 0 to 1 mg/mL. The AFC was measured after each addition of glucose solution to the working mixture in the cell with liquid. The phase shift of the acoustic signal was measured for each of the glucose concentrations at frequencies of 27.81 and 42.73 MHz. These frequencies were selected based on the analysis of the frequency dependences of the S_12_ parameter. The change in this parameter for the chosen frequencies was at maximum at the glucose concentration change. Figure 6 shows the time dependence of the change in glucose concentration in the measuring cell.

## 3. Results and Discussion

### 3.1. Influence of Adsorption of GOx Enzyme Molecules on the Surface Properties of a Langmuir DPPE Monolayer

The compression isotherms of the DPPE monolayer formed on the subphase in the absence of dissolved molecules of the GOx enzyme (1) and in their presence (2) are shown in Figure 1. Three regions, I-II, II-III, and III-IV, were observed on the compression isotherm of the DPPE monolayer. In these regions, the monolayer was in the gas, liquid, and condensed phases, respectively. The surface pressure in the DPPE monolayer is varied from 0.5 to 12.5 mN/m and from 12.5 to 61 mN/m in the liquid and condensed phases, respectively. The compression modulus of the DPPE monolayer without the GOx enzyme in the condensed phase was 133 mN/m and the *A*_0_ was 32.5 Å^2^. The addition of GOx enzyme molecules to the subphase led to a change in the shape of the compression isotherm. Before the start of the compression process of the DPPE monolayer, the molecules of the GOx enzyme were adsorbed at the water–air interface [67]. The adsorption of GOx molecules led to an insignificant increase in the surface pressure in the gas phase up to 2 mN/m. An increase in the number of molecules located on the water surface led to a shift in the compression isotherm towards larger areas. Thus, the phase transition points in the monolayer also shifted (Figure 1). For example, the phase transition point from the gas phase to the liquid phase for a DPPE monolayer without the enzyme corresponded to *A*_0_ = 65 Å^2^. The adsorption of GOx molecules at the boundary of the water surface led to a shift of this phase transition point (*A*_0_ = 140 Å^2^). In addition, an additional phase transition point (IIa) appeared in the section of the II-III compression isotherm. The phase transition point IIa corresponds to a surface pressure of 9 mN/m and an *A*_0_ value of 90 Å^2^. In region II–IIa of the compression isotherm of the DPPE monolayer formed on the subphase with dissolved GOx molecules, an increase in surface pressure from 2 mN/m to 9 mN/m was observed. The increase in the surface pressure in the DPPE monolayer is due to the onset of the process of interaction between the molecules of the GOx enzyme adsorbed on the water surface and the islands of DPPE molecules. In regions IIa-III of this compression isotherm, the surface pressure increased from 9 to 10 mN/m. The existence of such regions can be explained by the transition of the monolayer from the liquid-expanded phase to the liquid-condensed phase. Similar phase states were also observed in [68,69]. In this case, the presence of such regions was associated with a change in the structure of the monolayer and the formation of a multilayer film. However, in our case such a behavior of the compression isotherm can be associated with the interaction of hydrophobic parts of the enzyme molecules and hydrocarbon chains of lipid molecules [39]. At the same time, there was no tendency to form a multilayer structure on the water’s surface. Similar results were presented in [69]. Here, the presence of a plateau was explained by an increase in the strength of the electrostatic interaction between the head groups of fatty acid molecules during their deprotonation. In this regard, the existence of a plateau in the section of the IIa-III compression isotherm in our case can also be explained by an increase in the contribution from the electrostatic interaction between the GOx molecules and the head parts of the DPPE molecules to the intermolecular interaction in the monolayer. Further compression of the DPPE monolayer led to an increase in the surface pressure and *A*_0_ to 60 mN/m and 49 Å^2^, respectively. The compression modulus of the DPPE monolayer formed on the subphase with dissolved GOx molecules decreased to 87 mN/m. This indicates a decrease in the structural perfection of the monolayer.

### 3.2. Study of the Morphology of a Sensitive Coating Based on an LB DPPE Film with Immobilized GOx Enzyme Molecules

The study of the surface morphology was carried out using the method of atomic force microscopy on an NT-MDT Ntegra setup in a semi-contact mode with a scanning speed of one line of 0.65 Hz. An NT-MDT NSG10 series cantilever with a tip radius of <10 nm was used. The mathematical processing of images obtained in the study of the film surface morphology was carried out using the Gwyddion 2.61 software (Czech Metrology Institute, Brno, Czech Republic) [70,71]. Formula (2) was used to calculate the average film surface roughness:

(2) $$R_{a}=\frac{1}{N}\sum_{j=1}^{N}\left|r_{j}\right|,\;$$

where *R_a_* is the arithmetic mean deviation of the profile from the baseline (middle line of the profile), *N* is the number of points with the roughness parameter *R_a_* measured, and *r_j_* is the absolute deviation of the profile height value from the midline at each roughness measurement point.

**Figure 7:**
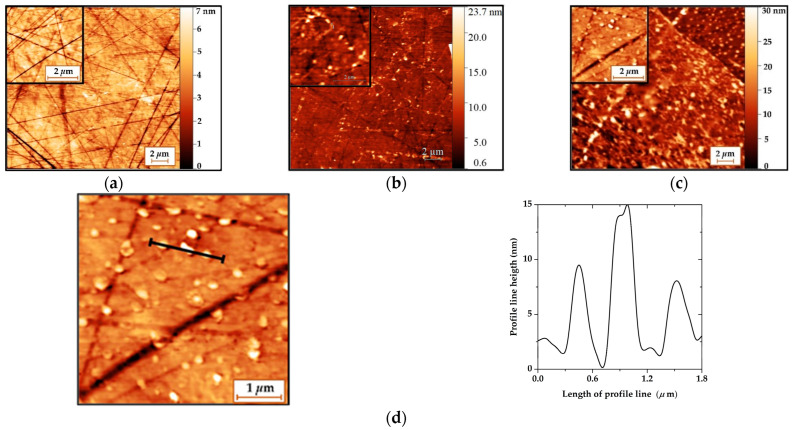
AFM images of the LiNbO_3_ plate surface without film (**a**), with a DPPE film (**b**), and with a DPPE film with immobilized GOx enzyme (**c**) and profile line of the surface of the DPPE film with immobilized GOx enzyme (**d**).

The images of the surface morphology of a clean LiNbO_3_ plate (Figure 7a), a LiNbO_3_ plate with a DPPE film (Figure 7b), and a LiNbO_3_ plate with a DPPE film with immobilized GOx enzyme molecules (Figure 7c) are presented in Figure 7. The surface of the LiNbO_3_ plate had a roughness of about 1 nm. The presence of stripes on the surface of the plate is associated with its polishing. The depth of the bands is ranged from 3 to 5 nm. The morphology of the plate surface is changed due to the deposition of an LB film of DPPE on it. The surface roughness increased up to 1.2 nm. This can be associated with the formation of defects (pores) in the film during its transfer and further drying.

**Figure 8:**
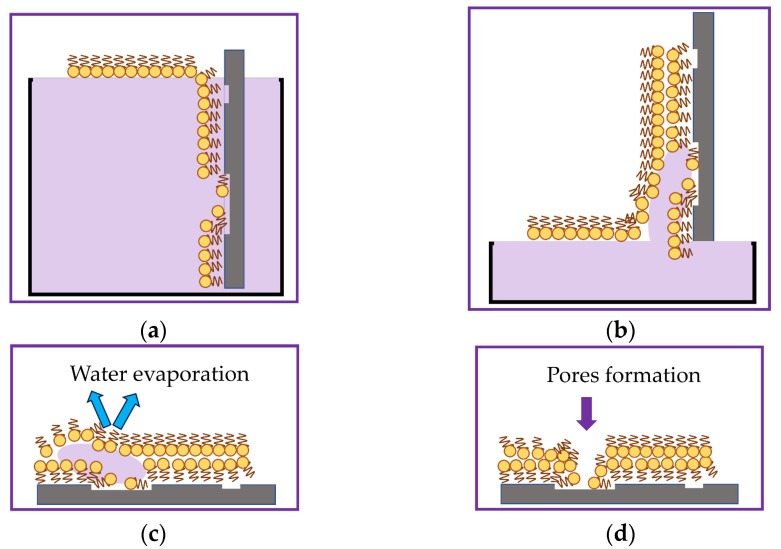
Schematic representation of the process of pore formation in a bilayer LB DPPE film on a lithium niobate substrate: formation of the first layer (**a**); capture of water molecules by capillary forces during the transfer of the second monolayer (**b**); modification of the film surface in places of accumulation of trapped water molecules (**c**); evaporation of water molecules and formation of pores in the film (**d**).

Schematically, the process of defect formation in the LB DPPE film is shown in Figure 8. The surface of the lithium niobate substrate is hydrophilic due to the presence of an uncompensated surface charge.

Its interaction with the water molecule dipole leads to better spreading of the water’s drop over the plate surface. During the formation of the first layer of the LB film, an interaction occurs between the hydrophilic substrate and DPPE molecules in the Langmuir monolayer, whose hydrophobic parts are oriented toward the substrate.

The presence of scratches and other defects on the surface of the lithium niobate substrate leads to breaks in the transferred monolayer (Figure 8a). In the process of applying the second layer of the film, water molecules are drawn into the gaps formed by capillary forces (Figure 8b). The evaporation of water during the drying of the film leads to the formation of pores in it (Figure 8c,d). With a large number of formed pores, a local change in the film thickness leads to a change in its roughness. The adsorption of GOx enzyme molecules by a Langmuir monolayer leads to a change in the morphology of the LB film formed on its basis. The film surface becomes more developed as the average film roughness increases from 1.2 to 5.5 nm. As is known, the size of the glucose oxidase enzyme molecule is 5.2 × 7.7 × 6.0 nm [72]. In the resulting LB film, aggregates with a height of 5 to 15 nm and an area of 0.03 µm^2^ to 0.1 µm^2^ are visible (Figure 7d). The heights of the aggregates in the LB film are comparable to the sizes of a single molecule of the GOx enzyme. In this connection, it can be concluded that immobilized molecules of the GOx enzyme are present in the created LB film.

### 3.3. Study of the Sensitivity of LB Film with DPPE and Immobilized GOx Enzyme to Glucose Solution

The frequency dependences of the *S*_12_ parameter of the manufactured acoustic DL with a DPPE LB film with an immobilized GOx enzyme were obtained in the absence of liquid in the cell, in the presence of distilled water, and in the presence of a glucose solution with various concentrations in the range from 0 to 1 mg/mL in the cell.

**Figure 9:**
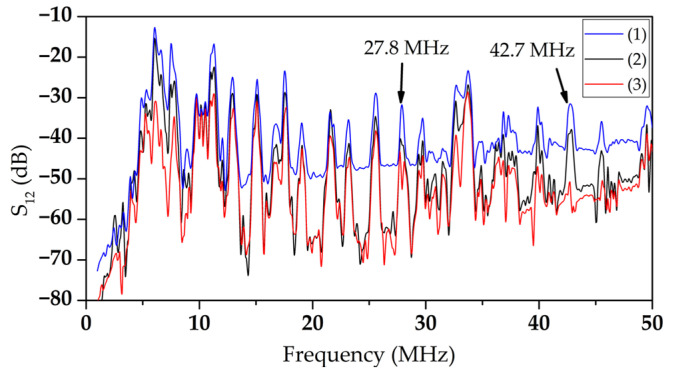
Frequency dependences of the *S*_12_ parameter of the manufactured acoustic DL with a DPPE LB film with immobilized GOx enzyme in the absence of liquid in the cell (1), in the presence of distilled water (2), and a glucose solution with a concentration of 0.3 mg/mL (3) in the cell.

As an example, the frequency dependences of the parameter *S*_12_ of the manufactured acoustic DL with a DPPE LB film with an immobilized GOx enzyme in the absence of liquid in the cell (1), in the presence of distilled water (2), and the glucose solution with a concentration of 0.3 mg/mL (3) in the cell are presented in Figure 9.

It can be seen that the addition of distilled water or glucose solution to the cell leads to an increase in insertion loss and a change in the view of AFC. It should be noted that some modes are characterized by strong attenuation in the presence of liquid. At the same time, other modes react insignificantly to the appearance of liquid. This is due to different polarizations of higher-order waves excited at different frequencies [73]. As a result of the analysis of the obtained results, modes with frequencies of 27.8 and 42.7 MHz were chosen as working ones. For these modes, the change in the S_12_ parameter was maximal with a change in the concentration of glucose, and they reacted weakly to the presence of distilled water.

**Figure 10:**
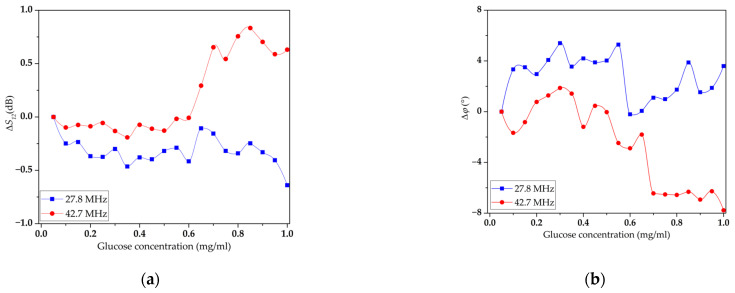
Concentration dependences of the S_12_ parameter (**a**) and phase shift (**b**) for modes at frequencies of 27.81 MHz (blue line) and 42.73 MHz (red line) for delay line without an LB film.

First, the effect of glucose solutions of various concentrations on the insertion loss and phase shift of the selected acoustic modes of the created DL without an LB film was studied. The corresponding concentration dependences of the S_12_ parameter and phase shift for the selected operating modes are shown in Figure 10. The measurement error of the S_12_ parameters and phase shift were 0.1 dB and 0.1^0^, respectively.

It can be seen that an increase in the concentration of glucose in distilled water leads to significant changes in the controlled parameters of the selected modes. The maximal changes in insertion loss and phase shift were 0.6 dB and 8°, respectively, for the mode at a frequency of 42.7 MHz. This can be explained by the insignificant influence of the conductivity of an aqueous glucose solution on piezoactive waves under these conditions. The experiments have shown that a change in the concentration of glucose in water in the range from 0 to 1 mg/mL leads to a change in its conductivity in the range from 0.04 to 0.1 μS/m. This range of conductivities is outside the range of applicability of the acoustoelectronic method [74].

**Figure 11:**
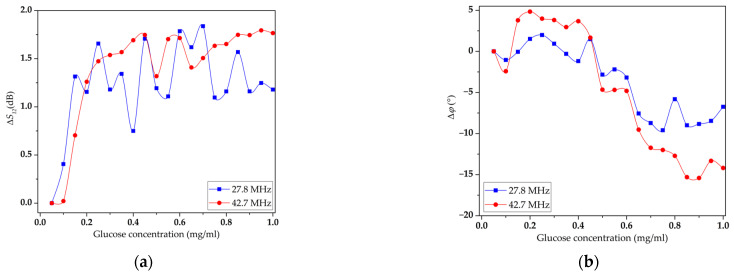
Concentration dependences of the *S*_12_ parameter (**a**) and phase shift (**b**) for modes with frequencies of 27.81 MHz (blue line) and 42.73 MHz (red line) for delay line coated with an LB DPPE film without GOx enzyme.

Then, the concentration dependences of the change in the *S*_12_ parameter and the phase shift were measured for the same modes in the case of the presence of an LB DPPE film without GOx on the DL surface. The resulting dependencies are shown in Figure 11. In this case, the maximum change in insertion loss for the 42.7 MHz mode increased from 0.6 to 1.6 dB, and the phase shift increased from 8 to 15°.

As shown earlier (Figure 3), the LB DPPE film has little effect on the *S*_12_ parameter for the selected modes with frequencies of 27.81 MHz and 42.73 MHz compared to the unloaded surface. Thus, the changes in the *S*_12_ parameter and the phase shift with an increase in the conductivity of the glucose on the surface of the sensor film without the GOx enzyme are associated with the interaction of glucose molecules directly with the LB film, its penetration into the pores, and an increase in the mass load. However, it should be noted that the changes in the measured parameters are still insignificant with increasing glucose concentrations in the solution.

**Figure 12:**
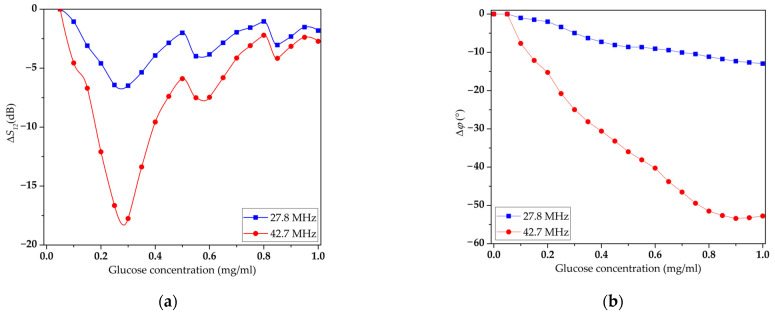
Concentration dependences of the S_12_ parameter (**a**) and phase shift (**b**) for modes with frequencies of 27.81 MHz (blue line) and 42.73 MHz (red line) for delay line coated with an LB DPPE film with immobilized GOx enzyme.

Finally, the concentration dependences of the changes in the *S*_12_ parameter and the phase shift were measured for the same modes in the presence of an LB film of DPPE with an immobilized GOx enzyme on the surface of the DL. The resulting dependencies are shown in Figure 12. It can be seen that as the glucose concentration increases, the insertion loss of the recorded modes increases, reaches a maximum, and then decreases. In this case, the phase of these modes decreases almost linearly over a wide range of glucose concentrations in the solution and reaches saturation at 0.9 mg/mL for the mode at a frequency of 42.7 MHz. The largest change in parameter *S*_12_ for the mode at a frequency of 42.7 MHz was 18 dB at a glucose concentration of 0.35 mg/mL. The largest phase shift, in this case, was 55° at a glucose concentration of 0.9 mg/mL for the same mode. Such behavior may be associated with a shift in the range of changes in the conductivity of the analyzed liquid in the range of 0.01–10 S/m [74]. In this case, it is possible to use acoustic methods to create glucose sensors. It should be noted that such a shift in the region of change in conductivity became possible by changing the concentration of the GOx enzyme in the LB DPPE film. Studies performed with other LB films, for example, based on stearic acid (SA) did not allow such an effect to be achieved. This may be due to the peculiarities of the interaction of the GOx enzyme with the molecules of the working solutions used. It was found that LB DPPE films modified with the GOx enzyme had a more developed surface than LB SA films with the same concentration of the enzyme. In addition, DPPE molecules have a higher affinity for GOx than SA molecules.

The shift in the conductivity region of the film and the increase in the range of its variation can be explained as follows. It is known that in the presence of the GOx enzyme the glucose molecule is oxidized to form gluconic acid and hydrogen peroxide [75]. During the formation of hydrogen peroxide, the GOx molecule participates in the transfer of an electron to an oxygen molecule. As a result of this process, the conductivity changes in the localization region of the GOx molecule. Since the GOx molecules were immobilized in the LB film located on the surface of the acoustic delay line, the conductivity changed mainly in the near-surface layer of the acoustic delay line. In this case, an increase in the number of GOx molecules involved in the catalytic decomposition of glucose led to a greater change in the conductivity of the near-surface layer of the acoustic DL. Exceeding the threshold value of conductivity in the near-surface layer led to the screening of the electric field of the acoustic wave and a decrease in its phase velocity. In turn, this process led to an increase in the insertion loss and a decrease in the amplitude response of the S_21_ acoustic wave at glucose concentrations exceeding 0.4 mg/mL.

As mentioned above, the acoustic mode at a frequency of 42.7 MHz has the highest sensitivity to changes in glucose concentration. It should be noted that for the acoustic mode at a frequency of 27.8 MHz, the S_12_ parameter increases from 1.75 to 7.5 dB. At the same time, the maximum phase shift for this acoustic mode did not change compared to the case of the absence of the enzyme in the LB film and amounted to 10°. Such differences in the responses of these waves are associated with different coefficients of their electromechanical coupling.

## 4. Conclusions

In this work, LB films of DPPE phospholipid molecules with immobilized GOx enzyme molecules were created. The effect of the adsorption of GOx enzyme molecules on the surface properties of a Langmuir DPPE monolayer was studied. A change in the shape of the monolayer compression isotherm was found upon the addition of GOx enzyme molecules to the subphase. This may be due to the interaction of the hydrophobic parts of the enzyme molecules and the hydrocarbon chains of lipid molecules. This effect leads to a decrease in the structural perfection of the monolayer. The adsorption of GOx enzyme molecules by a Langmuir monolayer leads to a change in the morphology of the LB film formed on its basis, and the film surface becomes more developed.

The sensory properties of the obtained LB film of DPPE phospholipid molecules with immobilized GOx enzyme molecules in a glucose solution of various concentrations were studied. It has been shown that the introduction of GOx enzyme molecules into an LB DPPE film leads to a shift in the conduction region of the solution toward higher values with an increasing glucose concentration. Such an effect made it possible to conclude for the first time that acoustic methods can be used to determine the concentration of glucose molecules in an aqueous solution. It was found that for an aqueous solution of glucose in the concentration range from 0 to 0.8 mg/mL, the phase response of the acoustic mode at a frequency of 42.7 MHz has a linear form and its maximum change was 55°. The maximum change in the S_12_ parameter for this mode was 18 dB for a glucose concentration in the working solution of 0.4 mg/mL.

Thus, it was concluded that it is possible to create an acoustoelectronic enzymatic glucose sensor based on an LB film of DPPE phospholipid molecules with an immobilized glucose oxidase enzyme. The main advantage of such sensors is their high selectivity and sensitivity to the detected molecules. At the same time, for the direct use of such sensors outside of laboratory conditions, it is necessary to solve several problems. In particular, this is the task of finding optimal storage conditions for a sensor with a film, which will ensure its durability and reusability. In addition, it is necessary to solve the problem of the possibility of restoring the sensory properties of enzyme films in case of a violation of their storage conditions. The study of the process of the formation of sensory coverage requires additional research. In particular, the relationship between the thickness of the sensor coating, the amount of enzyme immobilized in it, and the resulting phase and amplitude responses under the influence of glucose have not been sufficiently studied. An important issue is to increase the reproducibility of the sensory properties of such coatings. This issue is relevant due to the dependence of the sensitivity threshold of sensors of this type and the amount of enzyme immobilized in the created sensor coating.

It should also be noted that the measured concentration range of 0–0.9 mg/mL corresponds to the range of blood glucose concentration. The possibility of changing the glucose conductivity range depending on the concentration of the GOx enzyme in the LB film will make it possible to develop technological glucose sensors for its higher concentrations. Such sensors would be in demand in the food and pharmaceutical industries. The developed technology can become the basis for creating a new generation of acoustoelectronic biosensors in case of using other enzymatic reactions.

## Data Availability

No new data were created or analyzed in this study. Data sharing is not applicable to this article.
